# A Cross-Sectional Study of the Association of ABO Blood Group and Rh Type With Severity of COVID-19 Infection in a Tertiary Care Center of South India

**DOI:** 10.7759/cureus.25569

**Published:** 2022-06-01

**Authors:** Sherin Varghese, Anjali Shankar, Sawakar SS, Yogeshvar Gowda, Avin V

**Affiliations:** 1 General Medicine, Jagadguru Jayadeva Murugarajendra Medical College, Davangere, IND; 2 Internal Medicine, Jagadguru Jayadeva Murugarajendra Medical College, Davangere, IND; 3 Medical Intern, Jagadguru Jayadeva Murugarajendra Medical College, Davangere, IND

**Keywords:** multiorgan failure, intubation, mortality, inflammatory markers, covid-19 severity, rh type, blood group

## Abstract

Background

The novel coronavirus disease 2019 (COVID-19) was declared a pandemic that had affected 224 countries, causing >2.1 million deaths worldwide. The association of the different ABO blood groups with the risk and severity of COVID-19 infections has been speculated in many studies. This study aims to determine the incidence of COVID-19 infections among various blood groups and the association of ABO blood groups and Rh type with the severity of COVID-19 infection as well as with other outcome predictors of COVID-19 infection including neutrophil-to-lymphocyte ratio, D-dimer, ferritin, lactate dehydrogenase, and C-reactive protein.

Methodology

This was a retrospective study conducted among 150 serologically positive patients >18 years of age who underwent treatment in a district government hospital over two months. Patients were categorized into severity groups, and laboratory data were divided into those corresponding to severe disease and otherwise, in accordance with national guidelines. Appropriate statistical analysis was performed.

Results

The frequency of blood groups A, B, AB, and O was 30.7%, 29.4%, 13.7%, and 39%, respectively. There was a statistically significant number of patients belonging to non-O blood groups who developed a severe COVID-19 infection (group C) (p = 0.005). There was an increased risk of multiorgan failure (p = 0.035), non-invasive ventilation (p = 0.005), intubation, and mortality among non-O blood groups, and was the maximum for A blood group even after adjusting for age and pre-existing comorbidities. Increased D-dimer levels were noted in non-O blood groups (p = 0.037). No statistically significant association was found between Rh typing and the severity of COVID-19 infection.

Conclusions

Our ﬁndings provide evidence that individuals with non-O blood groups are susceptible to developing more severe COVID-19 infections and should take active preventive measures. Moreover, they should be cautiously monitored and treated once infected.

## Introduction

Coronavirus disease 2019 (COVID-19), caused by the novel severe acute respiratory syndrome coronavirus 2 (SARS-CoV-2), broke out in Wuhan, China, and was declared a global pandemic [1,2]. The disease exhibits a wide spectrum of severity. Recently, investigators from China reported that ABO type was strongly statistically associated with acquiring SARS-CoV-2 infection and survival following illness [3]. They suggested that host factors have an important role in predicting disease severity. While acquired comorbidities such as age, obesity, diabetes, and history of smoking are very likely related to clinical severity [4,5], it is also likely that genetic factors are relevant to the host thrombo-inflammatory response.

The ABO blood group system discovered by Landsteiner is a kind of cell identity system determined by the antigenic structure located on the surface of erythrocytes. The Rh system is another such grouping. A, B, AB, and O blood groups are qualitative characteristics, with phenotypic variations reflecting the genetic structure. Blood group antigens are genetically encoded, and these antigens may be a predisposing factor for some diseases and a protective factor for others. The carbohydrate moieties of the ABO blood groups are genetically inherited, and previous studies have suggested a correlation between ABO blood type and cardiovascular diseases, cancers, and even certain infections such as SARS coronavirus [6-8].

The protein on the surface of the SARS-CoV-2 virus called the receptor-binding domain (RBD), which is the part of the virus that attaches to the host cells, makes it an essential target for scientists trying to learn how the virus infects people. Assessing how the SARS-CoV-2 RBD interacted with respiratory and red blood cells in ABO and Rh blood types provides clinical clues as to how different people respond to COVID-19 infection. In this study, we sought to understand the association between SARS-CoV-2 infection/COVID-19 and blood type. We compared both ABO and Rh blood types.

## Materials and methods

This retrospective, cross-sectional study was conducted at the Chigateri General Hospital, Davangere, for two months (May and June 2021), with the aim to determine the association of ABO and RH blood type with the severity of COVID-19 infections. Approval from the Institutional Ethics Committee was obtained before commencing the study (approval number: JJMMC/IEC-40-2021). A total of 150 patients who were documented to be COVID-19 positive by reverse transcription polymerase chain reaction testing, who were above the age of 18 with available laboratory details required for the study, who had completed treatment in the study hospital, and who were discharged as per hospital protocol or suffered in-hospital mortality were included in this study. Patients admitted with other non-COVID-19 febrile illnesses, such as dengue, malaria, leptospirosis, and rickettsial fevers, and those discharged against medical advice or referred to a higher center were excluded.

Patients were categorized according to their disease severity as mild, moderate, and severe, as per the Ministry of Health and Family Welfare (MoHFW) guidelines [9]. All patients were managed as per the existing state guidelines. Mild cases were given symptomatic treatment. Moderate and severe patients were managed with remdesivir, intravenous (IV) corticosteroids, anticoagulation, oxygen supplementation, and mechanical ventilation on a case-by-case basis, along with supportive treatment. Poor disease outcomes were considered as death/intubation. Data regarding comorbidities considered to be risk factors for severe diseases were collected. Laboratory parameters corresponding to high-risk patients included the following: neutrophil/lymphocyte ratio of >17, D-dimer of >1,000 ng/mL, serum ferritin of >300 mg/mL, lactate dehydrogenase (LDH) of >450 U/L, and C-reactive protein (CRP) of >100 mg/L.

The patients’ laboratory records and clinical data were assessed and analyzed using SPSS version 27 (IBM Corp., Armonk, NY, USA). ABO and Rh-type blood groups were analyzed for their ability to predict susceptible individuals for COVID-19 infection and association with severity markers. The chi-square test and Fisher’s exact test were used to determine the association between the two variables. A p-value of 0.05 or less was deemed statistically significant.

## Results

This study included 150 patients with a mean age of 55.73 ± 13.50 years, with 89 (59.3%) male patients and 61 (40.7%) female patients. Among the patients analyzed, the majority belonged to the O-positive blood group (Table 1).

**Table 1:** Distribution of ABO groups and Rh types in the study population.

	A	B	AB	O	Total
Rh–	3 (6.5)	1 (9.1)	4 (9.1)	1 (2.0)	9 (6.0)
Rh+	43 (93.5)	10 (90.9)	40 (90.9)	48 (98.0)	141 (94.0)

Around half of the study population had diabetes mellitus (50.7%) or hypertension (49.3). There was no significant difference in the distribution of various comorbidities among the study population with different blood groups (Table 2).

**Table 2:** Baseline clinical characteristics of the study population analyzed among different blood groups. #Chi-square test. T2DM: type 2 diabetes mellitus; HTN: hypertension; IHD: ischemic heart disease; CVA: cerebrovascular accident; CKD: chronic kidney disease; OAD: obstructive airway disease

Blood groups		Total	A (%)	B (%)	AB (%)	O (%)	P-value	Rh+ (%)	Rh– (%)	P-value
Mean age (years)		55.73	57.24	54.41	51.27	56.49	0.507	55.63	57.22	0.733#
Gender	Male	89 (59.3)	24 (52.2)	22 (50.0)	7 (63.6)	36 (73.5)	0.076#	86 (61.0)	3 (33.3%)	0.160#
	Female	61 (40.7)	22 (47.8)	22 (50.0)	4 (36.4)	13 (26.5)		55 (39.0)	6 (66.7)	
T2DM	Yes	76 (50.7)	23 (50.0)	23 (52.3)	4 (36.4)	26 (53.1)	0.792#	71 (50.4)	5 (55.6)	1.000#
	No	74 (49.3)	23 (50.0)	21 (47.7)	7 (63.6)	23 (46.9)		70 (49.6)	4 (44.4)	
HTN	Yes	67 (44.7)	23 (50.0)	15 (34.1)	5 (45.5)	24 (49.0)	0.403#	63 (44.7)	4 (44.4)	1.000#
	No	83 (55.3)	23 (50.0)	29 (65.9)	6 (54.5)	25 (51.0)		78 (55.3)	5 (55.6)	
IHD	Yes	10 (6.7)	4 (8.7)	0 (0.0)	2 (18.2)	4 (8.2)	0.061#	9 (6.4)	1 (11.1)	0.472#
	No	140 (93.3)	42 (91.3)	44 (100.0)	9 (81.8)	45 (91.8)		132 (93.6)	8 (88.9)	
CVA	Yes	4 (2.7)	0 (0.0)	1 (2.3)	1 (9.1)	2 (4.1)	0.207#	4 (2.8)	0 (0.0)	1.000#
	No	146 (97.3)	46 (100.0)	43 (97.7)	10 (90.9)	47 (95.9)		137 (97.2)	9 (100.0)	
CKD	Yes	3 (2.0)	1 (2.2)	0(0.0)	0(0.0%)	2 (66.7%)	0.820#	3 (2.1)	0 (0.0)	1.000#
	No	147 (98.0)	45 (97.8)	44 (100.0)	11 (100.0)	47 (95.9)		138 (97.9)	9 (100.0)	
OAD	Yes	11 (7.3)	2 (4.3)	4 (9.1)	0 (0.0)	5 (10.2)	0.612#	11 (7.8%)	0 (0.0%)	1.000#
	No	139 (92.7)	44 (95.7)	40 (90.9)	11 (100.0)	0 (0.0)		130 (92.2)	9 (100.0)	

Severe COVID-19 infection was found to be more among patients with blood group A, while others had a higher frequency of moderate COVID-19 infections, and this difference was statistically significant (Table 3).

**Table 3:** COVID-19 severity among the different blood groups. *P-value of <0.05 is statistically significant; #Chi-square test. COVID-19: coronavirus disease 2019

		Severity			
		Mild	Moderate	Severe	P-value
Blood group	A	4 (8.7)	17 (37.0)	25 (54.3)	0.004*#
	B	2 (4.5)	25 (56.8)	17 (38.6)	
	AB	3 (27.3)	7 (63.6)	1 (9.1)	
	O	5 (10.2)	33 (67.3)	11 (22.4)	
Rh group	Rh+	14 (9.9)	75 (53.2)	52 (36.9)	0.432#
	Rh–	0 (0.0)	7 (8.5)	2 (3.7)	

Multiorgan dysfunction and the requirement for non-invasive ventilation (NIV) were found to be significantly higher among patients with blood group A, and the mean CT severity score was also found to be high among those with blood group A (Table 4).

**Table 4:** Association between various COVID-19 related complications and the ABO blood groups *P-value of <0.05 is statistically significant; #Chi-square test. NIV: non-invasive ventilation; COVID-19: coronavirus disease 2019

		Total	A	B	AB	O	P-value	Rh+	Rh–	P-value
Mortality	Yes	27 (18.0)	14 (30.4)	5 (11.4)	1 (9.1)	7 (14.3)	0.086#	25 (17.7)	2 (22.2)	0.665#
	No	123 (82.0)	32 (69.6)	39 (88.6)	10 (90.9)	42 (85.7)		116 (82.3)	7 (77.8)	
Intubation	Yes	18 (12.0)	7 (15.2)	5 (11.4)	1 (9.1)	5 (10.2)	0.927#	16 (11.3)	2 (22.2)	0.295#
	No	132 (88.0)	39 (84.8)	39 (88.6)	10 (90.9)	44 (89.8)		125 (88.7)	7 (77.8)	
NIV	Yes	37 (24.7)	18 (39.1)	13 (29.5)	1 (9.1)	5 (10.2)	0.004*#	35 (24.8)	2 (22.2)	1.000#
	No	113 (75.3)	28 (60.9)	31 (70.5)	10 (90.9)	44 (89.8)		106 (75.2)	7 (77.8)	
Multiorgan dysfunction	Yes	65 (43.3)	27 (58.7)	17 (38.6)	6 (54.5)	15 (30.6)	0.034*#	62 (44.0)	3 (33.3)	0.732#
	No	85 (56.7)	19 (41.3)	27 (61.4)	5 (45.5)	34 (69.4)		79 (56.0)	6 (66.7)	
CT severity score (mean score)		12.86 ± 6.45	15.79 ± 6.54	13.38 ± 5.78	10.88 ± 6.42	9.68 ± 5.53	0.000*#	12.88 ± 6.51	12.63 ± 5.78	0.915#

D dimer and N/L ratio were found to be high among patients with blood type A. There was a significant association between blood type and inflammatory markers, such as D dimer (p = 0.016), N/L ratio (p = 0.000), and total leukocyte count (p = 0.046) (Table 5).

**Table 5:** Laboratory parameters among the different blood groups. *P-value of <0.05 is statistically significant; #Chi-square test. LDH: lactate dehydrogenase; CRP: C-reactive protein; N/L ratio: neutrophil/lymphocyte ratio; TLC: total leucocyte count; PLT: platelet count

	Total	A	B	AB	O	P-value	Rh+	Rh–	P-value
D-dimer (>1,000 mg/mL)	44 (29.3)	18 (39.1)	17 (38.6)	1 (9.1)	8 (16.3)	0.016#	41 (29.1)	3 (33.3)	0.722#
Ferritin (>300 mg/mL)	96 (64.0)	33 (71.7)	28 (63.6)	6 (54.5)	29 (59.2)	0.542#	89 (63.1)	7 (77.8)	0.490#
LDH (>450 U/L)	67 (44.7)	25 (54.3)	21 (47.7)	4 (36.4)	17 (34.7)	0.238#	65 (46.1)	2 (22.2)	0.300#
CRP (>100 mg/L)	33 (22.0)	11 (23.9)	13 (29.5)	1 (9.1)	8 (16.3)	0.360#	29 (20.6)	4 (44.4)	0.108#
N/L ratio (>17)	50 (33.3)	23 (50.0)	19 (43.2)	1 (9.1)	7 (14.3)	0.000*#	47 (33.3)	3 (33.3)	1.000#
TLC (>12,000/µL)	42 (28.0)	16 (34.8)	6 (13.6)	5 (45.5)	15 (30.6)	0.046*#	39 (27.7)	3 (33.3)	0.711#
PLT (<1.5)	22 (14.7)	9 (19.6)	5 (11.4)	0 (0.0)	8 (16.3)	0.407#	20 (14.2)	2 (22.2)	0.621#

## Discussion

In this study, we examined the relationship between ABO blood groups and severity of inflammation, development of complications, and mortality among serologically positive COVID-19 patients. Around 18.0% of the study participants succumbed to death following COVID-19 infection in this study. Patients with blood type A had a higher frequency of mortality compared to other blood types. However, this difference was not statistically significant. Socio-demographic and clinical characteristics were similar in all the study groups, with no significant difference between groups (p >0.05). Hence, our study had an almost identical population except for blood type. It was interesting to note the presence of diabetes or hypertension among half the population in our study. In the study by Ray et al., diabetes mellitus was present among approximately 21-22% of the study population and chronic hypertension was present among 38-42% of the study population with COVID-19 infections [10]. The higher number of patients in our study can be attributed to the increased prevalence of diabetes mellitus in India. Mahmud et al. observed no statistically significant differences in the presenting features and comorbidities among patients with different blood groups [11].

According to the World Health Organization (WHO) report, among those who become symptomatic following COVID-19 infections, around 40% develop only mild and another 40% develop a moderate disease. Approximately 15% develop severe disease, and 5% have critical disease according to the WHO report [12]. Among our study participants, 9.3% had mild disease, 54.7% had moderate, and 36.0% had severe illness. In our study, there was a significant difference between the severity of COVID-19 infection and blood groups of the patients (p = 0.04), with blood type A showing a greater number of severe cases compared to other blood types. The above findings were similar to a study conducted by Guillon et al. among 265 COVID-19 patients, which showed that blood group A was more frequent in patients with severe COVID-19 infections compared to the normal population (p = 0.017) and blood group O was less frequent in severe COVID-19 patients who required prolonged hospitalization (P < 0.01) [13]. Zhou et al. reported a link between blood group A and increased susceptibility of thromboembolism, diabetes mellitus, and hypertension, suggesting high mortality and severity of COVID-19 in blood group A [14].

Our study also showed that there was significantly high incidence of multiorgan dysfunction and the need for NIV among COVID-19 patients with blood type A. However, the study conducted by Zietz et al. described that COVID-19 patients with blood type A had less severity of illness, less frequency of intubation, and less mortality compared to other blood types [15]. Ray et al. found that O and Rh- blood groups may be associated with a slightly lower risk for SARS-CoV-2 infection and severe COVID-19 illness [10]. Studies have shown that the risk for thrombotic events is significantly lower in people with blood group O compared to those with non-O blood groups, and COVID-19 infections are known to have increased risk for thrombosis, and blood group O might be lowering this risk [3,15,16].

The SARS-CoV that caused the SARS outbreak in Hong Kong infects cells that express ABH antigens according to the ABO phenotype of the individual. Pneumocytes, enterocytes of the small intestine, and the cells of the kidney distal tubular epithelium are known to be able to express ABH antigens, and SARS-CoV infections were found to be present in these cells [17,18]. Taking this into consideration, Guillon et al. conducted an experiment to determine if anti-histo-blood group antigens could block SARS-CoV entry into target cells and found that they inhibited the SARS-CoV S protein/angiotensin-converting enzyme 2-dependent adhesion. This may be the reason for the low risk of infection in blood group O individuals during the initial SARS outbreak [13]. Similar pathology may be seen in SARS-CoV-2 infection as well resulting in blood type O being slightly protective against COVID-19. May et al. [19], and Levi et al. [20] did not find any significant association between ABO blood groups and the outcome or risk of developing SARS-CoV-2 in their studies.

Considering various inflammatory laboratory markers, D-dimer, N/L ratio, and TLC were found to be significantly higher in those with blood type A. This may be because of the high prevalence of severe COVID-19 infections among patients with blood type A.

There are many risk factors, other than blood groups, which determine the severity of SARS-CoV-2 infection, and many are yet to be fully elucidated. More multicenter studies are required to prove the relationship between SARS-CoV-2 infection and blood groups.

Limitations

This was a single-center study with a limited sample size. The day of initiation of treatment from symptom onset was not considered as delayed treatment would contribute to increased mortality. There was not enough sample size to study the effect of Rh type on the severity of COVID-19 infection.

## Conclusions

Our results suggest that blood group A individuals are more prone to clinically severe infection with SARS-CoV-2 than others with increased chances of mortality. Hence, individuals with blood group A need to take active preventive measures and should be cautiously monitored and treated once infected.
